# An Assessment of Urea-Formaldehyde Fertilizer on the Diversity of Bacterial Communities in Onion and Sugar Beet

**DOI:** 10.1264/jsme2.ME13157

**Published:** 2014-05-30

**Authors:** Seishi Ikeda, Keijiro Suzuki, Makoto Kawahara, Masao Noshiro, Naokazu Takahashi

**Affiliations:** 1 Hokkaido Agricultural Research Center, National Agriculture and Food Research Organization, 9–4 Shinsei-minami, Memurocho, Kasai-gun, Hokkaido 082–0081, Japan; 2 Kitami Agricultural Experiment Station, Aza Yayoi, Kunneppu, Hokkaido 090–1496, Japan; 3 SUN AGRO Co., Ltd., 1470 Shiraoka, Shiraoka, Saitama 349–0218, Japan; 4 HOKKAIDO SUN AGRO Co., Ltd., Sapporo-daiichiseimei-building, Kita 3, Nishi 4, Chuo-ku, Sapporo, Hokkaido 060–0003, Japan

**Keywords:** bacterial community, nitrogen fertilizer, onion, sugar beet, urea-formaldehyde

## Abstract

The impact of a urea-formaldehyde (UF) fertilizer on bacterial diversity in onion bulbs and main roots of sugar beet were examined using a 16S rRNA gene clone library. The UF fertilizer markedly increased bacterial diversity in both plants. The results of principal coordinates analysis (PCoA) revealed that nearly 30% of the variance observed in bacterial diversity in both the onion and sugar beet was attributed to the fertilization conditions and also that the community structures in both plants shifted unidirectionally in response to the UF fertilizer.

Slow-release fertilizers have recently been gaining attention and have been reevaluated due to the recognition of their beneficial and pleiotropic properties (6). The use of a slow-release fertilizer offers economic and environmental benefits to farmers by saving time and reducing the labor involved with handling fertilizers and also prevents the loss of nitrogen by volatilization and leaching (1). Several types of slow-release fertilizers are currently available commercially (6). Of these, urea-formaldehyde (UF) is the oldest synthetic fertilizer and has been one of the most commonly used slow-release fertilizers worldwide.

UF fertilizers are mainly degraded in soil by microbes and decompose to urea and formaldehyde (13). Urea is used as a nitrogen source via the mineralization of urea to ammonium for a plant nutrient, while formaldehyde is generally incorporated immediately into the microbial C1 metabolic pathways in soil (12). Hadas *et al.* reported that the accelerated mineralization of UF in soils was associated with the continuous use of UF, and suggested that UF-degrading microbial populations may be increased in soil (7). However, only a few species of UF-degrading organisms have been identified to date (11, 12, 14). One of these studies identified *Agrobacterium tumefaciens* (currently proposed for reclassification as *Rhizobium radiobacter*) as an organism that degrades UF in field soils. This is an important concern for the future use of UF fertilizers because this particular bacterium is considered to be a potential plant pathogen (14). Hence, assessing the impact of UF fertilizers on the microbial communities associated with plants is of interest from the view point of plant microbiology. In the present study, the impacts of a UF fertilizer on onion- and sugar beet-associated bacteria were evaluated using clone library analysis.

The cultivar “Kitamomiji 2000” was used to assess the diversity of onion-associated bacteria. The experimental sites were two fields in local farms within the Hokkaido prefecture of Japan, designated site OA (0.5 a) (Kitami city, E143°54′N43°48′) and site OB (0.5 a) (Kunneppucho, Tokoro-gun, E143°44′N43°43′). The soils of both sites were classified as brown lowland soil. The preceding crop for fields OA and OB was onions because both fields were continuous cropping fields for onions. Prior to the planting of onion seedlings, the test fields were dressed with fertilizers by broadcasting on 21 April and 6 May 2010 for sites OA and OB, respectively. The control fields were dressed with the commercial fertilizer S006 (120, 240, 72, and 36 kg of N, P_2_O_5_, K_2_O, and MgO ha^−1^, respectively; Hokuren Fertilizer Corporation, Sapporo, Japan) for site OA and S245 (120, 240, 50, and 30 kg of N, P_2_O_5_, K_2_O, and MgO ha^−1^, respectively; Hokuren Fertilizer Corporation) for site OB. The nitrogen sources in the control fertilizers were ammonium sulfate (70% and 87.5% of total nitrogen for S006 and S245, respectively) and nitrate nitrogen (30.0% and 12.5% for S006 and S245, respectively). The OA and OB sites were also dressed with the UF-containing commercial fertilizer UFS085 (120, 216, 60, and 24 kg of N, P_2_O_5_, K_2_O, and MgO ha^−1^, respectively; HOKKAIDO SUN AGRO, Sapporo, Japan) as for the UF fields. The UF fraction relative to the total nitrogen level in the UF fertilizer was 30% (40 kg ha^−1^), and the remaining 70% was ammonium sulfate. The UF fraction was comprised of condensation products derived from a reaction between 2 moles of urea and 1 mol of formaldehyde. Total nitrogen, water insoluble nitrogen, and the activity index for UF were 41.3%, 11.6% and 58%, respectively. Onion seedlings that had been grown in a greenhouse were planted on 5 May and 18 May 2010 at sites OA and OB, respectively. The widths of the rows were 30.6 and 28.5 cm for Kitami city and Kunneppucho, respectively, while the spacings between plants in a row were 11.7 cm and 11.3 cm. Planting densities for Kitami city and Kunneppucho were 27999 and 31013 plants 10 a^−1^, respectively. The average temperature and precipitation during the growth period (from May to August, 2010) were 18.2°C and 393.5 mm, respectively, for Kitami city and 17.3°C and 421.5 mm, respectively, for Oketocho (E143°38.6′N43°42.4′), which was the closest measurement point of the AMeDAS (Automatic Meteorological Data Acquisition System) to Kunneppucho. Three plants that showed a representative growth pattern were sampled from each test field on 23 August and 30 August 2010 for sites OA and OB, respectively.

The cultivar “Kachimaru” was used to assess the diversity of sugar beet-associated bacteria. The experimental sites were also in local farms in the Hokkaido prefecture of Japan and designated site SA (50 a) (Nakasatsunai village, Kasai gun, E143°08′N42°42′) and site SB (1 ha) (Obihiro city, E143°12′N42°55′). The preceding crops for SA and SB were Chinese yam and sugar beet, respectively. The soils of both sites were classified as low-humic andosol. Prior to the sowing of sugar beet seeds, sites SA and SB were dressed with commercial fertilizers by broadcasting on 1 May and 6 May 2010, respectively. The control fields were dressed with a local brand of commercial fertilizer (160, 300, 160, and 70 kg of N, P_2_O_5_, K_2_O, and MgO ha^−1^, respectively) for site SA, and BBS171 (154, 238, 154, and 70 kg of N, P_2_O_5_, K_2_O and MgO ha^−1^, respectively; Hokuren Fertilizer Corporation) for site SB. The nitrogen sources in the control fertilizers consisted of ammonium sulfate (67.0% and 54.5% of total nitrogen for the local brand fertilizer and BBS171, respectively), nitrate nitrogen (33.0% and 18.2% for the local brand fertilizer and BBS171, respectively), and urea (27.3% for BBS171). The UF areas of sites SA and SB were dressed with UFS 605 (160, 200, 50, and 30 kg of N, P_2_O_5_, K_2_O, and MgO ha^−1^, respectively; HOKKAIDO SUN AGRO). The UF fraction relative to the total nitrogen level in the UF fertilizer was 25% (40 kg ha^−1^), and the rest of the nitrogen components was composed of ammonium sulfate (56.2%) and nitrate nitrogen (18.8%). The chemical characteristics of the UF fraction were identical to those described for UFS085. The width of the rows and the spacing between the plants were 66.0 cm and 16 cm, respectively, for both Nakasatsunai village and Obihiro city. Planting densities were 9,469 plants 10 a^−1^ for both Nakasatsunai village and Obihiro city. The average temperature and precipitation during the growth period (from May to September, 2010) were 16.1°C and 662 mm, respectively, for Nakasatsunai village and 18.0°C and 643 mm, respectively, for Obihiro city. Three plants showing representative growth patterns were again sampled from each of the control and UF fertilization areas for sites SA and SB on 7 October 2010. Soil samples were collected prior to the dressing fertilizers in both the onion and sugar beet fields. The chemical characteristics of the soil samples were analyzed by the Tokachi Nokyoren Agricultural Research Institute (Obihiro, Japan) (Table 1). The soil sample for SA was lost due to a technical reason during the analysis.

Onion bulbs and the main roots of the sugar beet were washed with sterilized water, and stored at −30°C until required for DNA extraction. Dead portions of the onion bulb surface tissue were discarded. Plant materials were individually processed for bacterial cell enrichment, DNA extraction, and PCR amplification of the 16S rRNA gene as described previously (16). Briefly, for each plant material, approximately 50 g of the onion bulb or 300 g of the main root of the sugar beet were used to prepare bacterial cells (including epiphytes and endophytes). The PCR products derived from three plants for each of the control and UF fertilizations in the same experiment sites were combined into a composite sample that was then resolved by 1% agarose gel electrophoresis in TBE (89 mM Tris-Borate, 0.2 mM EDTA) buffer. The amplicons of a predicted size (approximately 1,500 bp) were cloned into a plasmid and sequenced. Sequence editing and community analyses were conducted as described previously (16). The sequence data for the clone libraries in the present study were deposited in public databases under the accession numbers shown in the supplementary Table 1.

Statistical analyses revealed that larger numbers of genera, OTUs, and singletons were observed for all libraries derived from UF fields (UF libraries) than those from the corresponding control fields for both the onion and sugar beet (Table 2). The Shannon and Simpson indexes were also higher for the UF libraries than the control libraries for both the onion and sugar beet.

Phylogenetic analyses revealed that the relative abundances of two genera of *Alphaproteobacteria* (*Methylobacterium* and *Sphingomonas*) were significantly higher in the UF libraries than in the control libraries for both OA and OB sites (Table 3-A). Similar results were observed for the relative abundances of the genus *Burkholderia* and phylum *Actinobacteria*. The results of clustering analyses identified OTUs that corresponded to two genera *Methylobacterium* and *Sphingomonas* in onion bulbs (OAP 1, OAP2, OAP3, OAP4, and OAP17 in Supplementary Fig. 1-A). The abundance and intragenus diversity of the genus *Methylobacterium* were higher with UF fertilization than with control fertilization. The abundance for an OTU relating to *Phyllobacterium myrsinacearum* was markedly high in site OA, which produced a higher yield than that of site OB (Supplementary Table 2). This was an interesting result because *P. myrsinacearum* is a well-known PGPR (3). In the case of sugar beet-associated bacteria, phylogenetic analyses revealed that the relative abundance of the genus *Aquicella* was slightly higher in the UF libraries than in the control libraries for both the SA and SB sites (Table 3-B). An OTU closely related to *A. tumefaciens* was identified in onion bulbs grown using the UF fertilizer for sites OA and OB (OTU OAP10 in Supplementary Table 2), while no OTU belonging to *Agrobacterium* sp. was detected in the sugar beet. The abundance for this OTU was small and did not significantly differ between the control and UF fertilization areas. The representative sequence of this OTU was not identical to *A. tumefaciens*. Based on these results, the potential risk of UF of enhancing the multiplication of certain plant pathogenic bacteria such as *A. tumefaciens* appears to be low. However, *A. tumefaciens* is an important plant pathogen in a wide variety of plant species (14); therefore, this bacterial group needs to be examined in more detail in future studies in association with UF fertilization.

The results of principal coordinates analyses indicated that the community structures of onion- and sugar beet-associated bacteria were mainly affected by the field locations, as explained by PC1 in Fig. 1. However, these results also revealed that UF fertilization had a markedly affected the community structures of plant-associated bacteria, as explained by PC2 in Fig. 1.

The impact of fertilization management on a microbial community in a phytosphere has been examined previously (2, 4, 8, 9, 15). In these earlier studies, a series of diversity indexes had no significant impact on bacterial communities. The results of the present study clearly indicated that onion-and sugar beet-associated bacteria were more diverse with UF fertilization than with the corresponding control fertilization (Table 2). The increase in bacterial diversity was mainly observed at the species level, most notably in the case of *Alphaproteobacteria* and *Gammaproteobacteria*, for onion-and sugar beet-associated microbes (Supplementary Fig. 1).

Bacteria belonging to *Methylobacterium*, *Sphingomonas*, and *Burkholderia* can metabolize C1 compounds (5, 16, 17, 19). This metabolic ability may contribute to the proliferation of these bacterial groups with UF fertilization because C1 compounds such as formaldehyde and formate can be generated in a rhizosphere during the degradation of UF in soil (13). An increase in these genera in a phytosphere may be an advantage of using UF fertilizers because these bacterial groups are also known to contribute to plant health by promoting plant growth and protecting against plant diseases (10, 18, 19).

The results of the present and previous studies by our group (9) clearly demonstrated that fertilization practices should be recognized as one of the dominant forces that can shape microbial community structures in a phytosphere, and also suggest that a UF fertilizer may be used as a driving force to manipulate a bacterial community in plants. Since each type of fertilizer possesses unique physical and chemical structures (6), the pleiotropic impact of these compounds on plant-associated microbes as well as agronomic products should continue to be carefully evaluated in future studies. This in turn may help to build a more sophisticated and sustainable agricultural system.

## Supplementary Information



## Figures and Tables

**Fig. 1:**
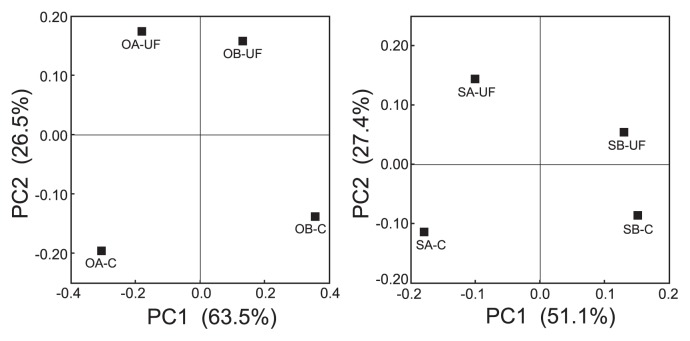
Principal coordinates analyses of the 16S rRNA gene sequences in clone libraries for onion- and sugar beet-associated bacteria. The ordinations were constructed for onion- and sugar beet libraries (A and B, respectively) using UniFrac distances weighted by the relative abundances. OA, OB, SA, and SB denote the experimental field sites. C and UF indicate conventional and UF fertilization, respectively.

**Table 1:** Chemical characteristics of soil samples

Soil characteristics	Onion		Sugar beet^a^

	Site OA	Site OB	Site SB
pH (H_2_O)	6.4	5.9	6.0
Organic C (kg kg^−1^)	0.012	0.016	0.032
Autoclave-extractable nitrogen (mg kg^−1^)	45.0	60.0	39.0
Truog P (mg kg^−1^)	1574.0	1006.0	92.0
Ex-K_2_O (mg kg^−1^)	666.0	640.0	177.0

a A soil sample for the SA field due to a technical reason.

**Table 2:** Characteristics of clone libraries of onion- and sugar beet-associated bacteria

Libraries	Onion				Sugar beet			
	
	Site OA		Site OB		Site SA		Site SB	
			
	C^a^	UF^a^	C	UF	C	UF	C	UF
Statistics								
No. of sequences	87	74	90	76	84	89	80	89
No. of Genera^b^	5	8	15	16	22	26	19	22
No. of OTUs^c^	12	22	16	25	48	65	43	55
No. of singletons	9	12	8	16	34	51	29	36
Library coverage (%)^d^								
Diversity indexes	89.7	83.8	91.1	78.9	59.5	42.7	63.8	59.6
Shannon index (*H*′)	1.0	2.3	2.0	2.5	3.4	4.0	3.5	3.8
Simpson index (1/*D*)	1.7	5.5	4.8	6.9	17.4	93.2	32.9	66.4

a C and UF indicate conventional and UF fertilization, respectively.

b Number of genera based on 16S rRNA gene sequences classified by RDPII.

c OTUs were defined at 97% sequence identity.

d *C_X_*=(*n*/*N*), where *n_x_* is the number of singletons that are encountered only once in a library and *N* is the total number of clones.

**Table 3-A:** Phylogenetic composition of onion-associated bacteria

Phylogeentic composition^a^	Libraries (%)			

	Site OA		Site OB	
	
	C^b^	UF^b^	C	UF
Proteobacteria	87.4	83.8	86.7	56.6**
Alphaproteobacteria	81.6	73.0	7.8	38.2**
*Methylobacterium*	2.3	13.5*	—	11.8**
*Phyllobacterium*	75.9	40.5**	3.3	3.9
*Sphingomonas*	2.3	12.2*	2.2	17.1**
Other genera	1.1	6.8	2.3	5.4
Betaproteobacteria	4.6	6.8	7.8	5.3
*Burkholderia*	—	4.1	—	3.9
Other genera	4.6	2.7	7.8	1.4
Gammaproteobacteria	1.1	4.1	71.1	13.2**
*Enterobacter*	—	—	38.9	7.9**
*Pseudomonas*	—	—	17.8	—**
Other genera	1.1	4.1	14.4	5.3
Firmicutes	10.3	9.5	13.3	35.5**
*Staphylococcus*	9.2	6.8	11.1	34.2**
Other genera	1.1	2.7	2.2	1.3
Actinobacteria	2.3	6.8	—	7.9**

a 16S rRNA gene sequences were classified by RDPII. The compositions of genera are shown for only dominant groups.

b C and UF indicate the conventional and UF fertilization, respectively.

* and ** indicate significance at the 1 and 5% levels (*P* < 0.01 and *P* < 0.05), respectively, calculated with the Library Compare of RDP II, between the conventional and UF fertilizations (C and UF, respectively).

**Table 3-B:** Phylogenetic composition of sugar beet-associated bacteria

Phylogeentic composition^a^	Libraries (%)			

	Site SA		Site SB	
	
	C^b^	UF^b^	C	UF
Proteobacteria	71.4	67.4	76.3	69.7
Alphaproteobacteria	23.8	29.2	48.8	36.0
Unclassified Bradyrhizobiaceae	8.3	10.1	12.5	4.5
Other genera	15.5	19.1	36.3	31.5
Betaproteobacteria	3.6	6.7	13.8	5.6
*Polaromonas*	1.2	—	10.0	—**
Other genera	2.4	6.7	3.8	5.6
Gammaproteobacteria	40.5	29.2	8.8	23.6**
*Aquicella*	—	4.5	1.3	10.1*
*Pseudomonas*	22.6	4.5**	—	—
Unclassified				
Gammaproteobacteria	8.3	10.1	7.5	10.1
Other genera	9.6	10.1	—	3.4
Deltaproteobacteria	—	—	—	1.1
Unclassified Proteobacteria	3.6	2.2	5.0	3.4
Planctomycetes	10.7	11.2	11.3	15.7
Firmicutes	4.8	5.6	2.5	1.1
Actinobacteria	2.4	1.1	2.5	5.6
Verrucomicrobia	3.6	7.9	3.8	6.7
Spartobacteria genera incertae sedis	2.4	4.5	3.8	6.7
Other genera	1.2	3.4	—	—
Acidobacteria	1.2	—	—	—
Deinococcus-Thermus	—	1.1	—	—
Unclassified Bacteria	6.0	5.6	3.8	1.1

a 16S rRNA gene sequences were classified by RDPII. The compositions of genera are shown for only dominant groups.

b C and UF indicate conventional and UF fertilization, respectively.

* and ** indicate significance at the 1 and 5% levels (*P* < 0.01 and *P* < 0.05), respectively, calculated with the Library Compare of RDP II, between the conventional and UF fertilizations (C and UF, respectively).
