# Supplementary material for: An Assessment of Urea-Formaldehyde Fertilizer on the Diversity of Bacterial Communities in Onion and Sugar Beet
Source: Microbes Environ. 2014 May 30;29(2):231–4. doi: 10.1264/jsme2.ME13157 (PMC4103532; doi:10.1264/jsme2.ME13157)
Supplement: Supplementary file 1 [file 29_231_s1.pdf]

Supplementary materials

An assessment of Urea-Formaldehyde Fertilizer on the Diversity of Bacterial Communities in Onion and Sugar beet

SEISHI IKEDA[1]*, KEIJIRO SUZUKI[2,3], MAKOTO KAWAHARA[4],

MASAO NOSHIRO[5], and NAOKAZU TAKAHASHI[4]

[1]*Hokkaido Agricultural Research Center, National Agriculture and Food Research Organization, 9-4 Shinsei-minami, Memuro-cho, Kasai-gun, Hokkaido 082-0081, Japan;* [2]*Kitami Agricultural Experiment Station, Aza Yayoi, Kunneppu, Hokkaido 090-1496, Japan;* [3]*Present address: Central Agricultural Experiment Station, Naganuma, Hokkaido 069-1395, Japan;* [4]*SUN AGRO Co., Ltd., 1470 Shiraoka, Shiraoka, Saitama 349-0218, Japan; and* [5]*HOKKAIDO SUN AGRO Co., Ltd., Sapporo-daiichiseimei-building, Kita 3, Nishi 4, Chuo-ku, Sapporo, Hokkaido 060-0003, Japan*

*Corresponding author. E-mail: sikeda67@affrc.go.jp; Tel: +81-155-62-9276; Fax: +81-155-61-2127.

Topic of the manuscript: ecology of plant-associated bacteria

Running title: Impacts of a UF fertilizer on plant bacteria

Supplementary Table 1. Accession numbers for clone libraries used in the present study

Accession numbers	Libraries
AB810269-AB810355	control library for site OA
AB810356-AB810445	control library for site OB
AB810446-AB810519	UF library for site OA
AB810520-AB810595	UF library for site OB
AB810596-AB810679	control library for site SA
AB810680-AB810759	control library for site SB
AB810760-AB810848	UF library for site SA
AB810849-AB810937	UF library for site SB

Supplementary Table 2. Yields data in the present stu

		Yields (t/ha)	
Crops	Locations	C[a]	UF[a]
Onion	OA (Kitami city)	63.6	67.1
Onion	OB (Kunneppucho)	52.9	55.2
Sugar beet	SA (Nakasatsunai village)	57.9	61.4
Sugar beet	SB (Obihiro city)	32.4	38.6

[a]C and UF indicate the conventional and UF fertilization, respectively.

A

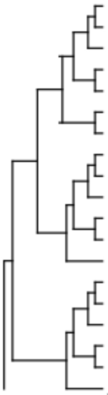

	Libraries (%)						
	Site OA		Site OB				
OTUs	C	UF	C	UF	Closest known species	Acc. No.	Identity (%)
OAP1	1.1	5.4	-	1.3	*Methylobacterium brachiatum*	AB698745	100
OAP2	-	1.4	-	-	*Methylobacterium hispanicum*	HE800584	99
OAP3	-	0.0	-	1.3	*Methylobacterium adhaesivum*	AB698724	99
OAP4	-	5.4*	-	5.3*	*Methylobacterium aquaticum*	AB698676	99
OAP5	1.1	1.4	-	3.9	*Methylobacterium populi*	JX993406	100
OAP6	1.1	-	-	-	*Bradyrhizobium elkanii*	JQ911628	100
OAP7	-	1.4	1.1	-	*Bosea vestrisii*	JX974340	99
OAP8	-	-	-	1.3	*Rhizobium huautlense*	AM237359	99
OAP9	-	1.4	-	-	*Rhizobium etli*	AM921623	99
OAP10	-	2.7	-	2.6	*Agrobacterium tumefaciens*	AB680310	99
OAP11	-	-	1.1	-	*Aurantimonas ureilytica*	AB682441	99
OAP12	-	1.4	-	-	*Aurantimonas altamirensis*	AB682666	100
OAP13	75.9	40.5**	3.3	3.9	*Phyllobacterium myrsinacearum*	JX512224	100
OAP14	-	-	1.1	-	*Sphingomonas mali*	AB740933	98
OAP15	-	1.4	-	1.3	*Sphingomonas aquatilis*	AB681116	99
OAP16	-	1.4	-	-	*Sphingomonas trueperi*	JQ579645	99
OAP17	1.1	9.5*	1.1	14.5*	*Sphingomonas sanguinis*	JQ660132	99
OAP18	1.1	-	-	-	*Sphingomonas yunnanensis*	HM069130	98
OAP19	-	-	-	1.3	*Sphingomonas humi*	JX094162	98

B

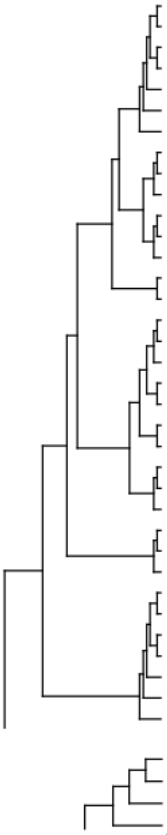

	Libraries (%)						
	Site SA		Site SB				
OTUs	C	UF	C	UF	Closest known species	Acc. No.	Identity (%)
Gammaproteobacteria							
SGP1	-	-	1.3	1.1	*Aquicella siphonis*	NR_025764	95
SGP2	-	1.1	-	-	*Aquicella siphonis*	AY359284	93
SGP3	-	1.1	-	2.2	*Aquicella siphonis*	AY359284	92
SGP4	-	1.1	-	-	*Aquicella siphonis*	AY359284	92
SGP5	-	1.1	-	-	*Aquicella siphonis*	NR_025764	92
SGP6	-	-	-	1.1	*Aquicella siphonis*	NR_025764	91
SGP7	-	-	-	5.6*	*Aquicella siphonis*	NR_025764	88
SGP8	-	-	1.3	-	*Legionella pneumophila*	FR799704	88
SGP9	-	-	1.3	-	*Legionella drancourtii*	NR_026335	87
SGP10	-	1.1	-	1.1	*Ectothiorhodospira shaposhnikovii*	FR733667	87
SGP11	-	-	-	1.1	*Legionella oakridgensis*	JX827100	86
SGP12	1.2	2.2	-	-	*Legionella oakridgensis*	JX827100	86
SGP13	-	-	1.3	2.2	*Legionella gresilensis*	NR_028744	86
SGP14	-	-	1.3	-	*Legionella nautarum*	NR_044961	86
SGP15	-	1.1	-	-	*Coxiella burnetii*	D89792	83
SGP16	-	-	1.3	-	*Methylobacter whittenburyi*	NR_029242	89
SGP17	-	-	-	2.2	*Ectothiorhodosinus mongolicus*	NR_042831	89
SGP18	-	1.1	-	-	*Legionella brunensis*	NR_026520	87
SGP19	-	-	1.3	-	*Legionella rowbothamii*	NR_036804	85
SGP20	-	1.1	-	-	*Lysobacter daejeonensis*	AB682404	85
SGP21	-	-	-	1.1	*Coxiella burnetii*	CP000890	86
SGP22	1.2	1.1	-	-	*Coxiella burnetii*	CP000890	87
SGP23	2.4	-	-	-	*Rickettsiella tipulae*	EU180598	85
SGP24	-	1.1	-	-	*Rickettsiella tipulae*	EU180598	84
SGP25	-	-	-	1.1	*Rickettsiella grylli*	U97547	89
SGP26	2.4	-	-	1.1	*Coxiella burnetii*	CP000890	90
SGP27	2.4	-	-	-	*Coxiella burnetii*	CP000890	89
SGP28	-	1.1	-	-	*Coxiella burnetii*	CP000890	89
SGP29	2.4	2.2	-	-	*Cedecea davisae*	AB682275	99
SGP30	-	3.4	-	-	*Pectobacterium carotovorum*	JX196705	100
SGP31	1.2	2.2	-	-	*Erwinia persicina*	EF693791	99
SGP32	2.4	1.1	-	-	*Pantoea agglomerans*	HE647624	99
SGP33	-	-	-	1.1	*Serratia grimesii*	JQ008935	99
SGP34	-	-	-	1.1	*Klebsiella oxytoca*	HQ683968	91
SGP35	22.6	4.5**	-	1.1	*Pseudomonas salomonii*	JX840381	100
Bradyrhizobiaceae							
SAP1	-	-	1.3	-	*Rhodopseudomonas rhenobacensis*	FN796846	94
SAP2	7.1	6.7	10.0	4.5	*Rhodopseudomonas rhenobacensis*	FN796846	98
SAP3	2.4	2.2	2.5	2.2	*Bradyrhizobium elkanii*	JQ911631	99
SAP4	-	1.1	-	-	*Rhodopseudomonas rhenobacensis*	FN796846	95

Supplementary Figure 1. The phylogenetic distribution of OTUs based on 16S rRNA gene sequences of the clone libraries for onion- and sugar beet-associated bacteria. The dendrogram indicates the phylogenetic relationships among the representative sequences of OTUs (defined by ≥97% identity). The table indicates the relative abundance of clones or isolates belonging to each OTU in each libraryand the results of a BLAST search using representative sequences. Panel A, Alphaproteobacteria among the onion-associated bacteria; panel B, Alpha- and Gammaproteobacteria among the sugar beet-associated bacteria. * and ** indicate statistical significance calculated using the Fisher's exact test at the 1 and 5% levels ($P< 0.01$ and $P< 0.05$), respectively, between conventional and UF fertilization conditions (C and UF, respectively).
